# Evaluation of Antioxidant and Antibacterial Activities of White Mulberry (*Morus alba* L.) Fruit Extracts

**DOI:** 10.3390/plants10122736

**Published:** 2021-12-12

**Authors:** Sureeporn Suriyaprom, Thida Kaewkod, Itthayakorn Promputtha, Mickaël Desvaux, Yingmanee Tragoolpua

**Affiliations:** 1 Department of Biology, Faculty of Science, Chiang Mai University, Chiang Mai 50200, Thailand; suriyaprom.sureeporn@gmail.com (S.S.); tda007suju@gmail.com (T.K.); ppam118@gmail.com (I.P.); 2 Graduate School, Chiang Mai University, Chiang Mai 50200, Thailand; 3 UMR454 MEDiS, INRAE, Université Clermont Auvergne, 63000 Clermont-Ferrand, France; mickael.desvaux@inrae.fr; 4 Research Center in Bioresources for Agriculture, Industry, and Medicine, Faculty of Science, Chiang Mai University, Chiang Mai 50200, Thailand

**Keywords:** anti-adhesion, antioxidation, inhibition, pathogenic enteric bacteria, white mulberry

## Abstract

The fruit of mulberry trees (*Morus* sp.), mulberries, are traditionally utilised as a nutritional food and provide health benefits as well as skin nourishment in Thailand. White mulberries (*Morus alba* L.) from Chiang Mai and Mae Hong Son provinces were evaluated for their antioxidant and antibacterial activities. The antioxidant activities as well as the total phenolic, flavonoid and anthocyanin content of the aqueous and ethanolic extracts were determined using 2,2-diphenyl-1-picrylhydrazyl (DPPH), 2,2′-azinobis-(3-ethylbenzothiazolin-6-sulfonic acid) (ABTS) and ferric reducing antioxidant power (FRAP) assays. The aqueous extracts of mulberries exhibited the highest antioxidant activity, which was associated with a higher phenolic and anthocyanin content. In testing the potent antibacterial activity against *Escherichia coli*, *Salmonella* Typhi, *Shigella dysenteriae*, *Staphylococcus aureus* and *Vibrio cholerae*, the mulberry extracts proved to be quite efficient, especially following water extraction. Time-kill and antibacterial adhesion assays further indicated that aqueous mulberry extracts could inhibit bacterial growth and prevent adhesions of pathogenic enteric bacteria on intestinal epithelial cells. It thus appears that mulberries can potentially be consumed as a good source of antioxidants, containing antimicrobial properties against some pathogenic bacteria which cause gastrointestinal tract infections.

## 1. Introduction

Pathogenic enteric bacteria are the main causative agents of human gastrointestinal tract infections and remain a prominent public health concern worldwide [1]. They can cause serious infectious diseases such as cholera with *Vibrio cholerae*, dysentery with *Shigella* sp. or salmonellosis with *Salmonella* sp. [2]. While rehydration therapy coupled with antibiotic treatment can be prescribed during the illness, especially in the case of severe diarrhoea, incautious antibiotic treatment can lead to the development of multi-drug resistant bacteria and/or chronic carriers [3]. The distribution of antimicrobial resistance is of global concern, and natural compounds with high pharmacological activity and low cytotoxicity can be an alternative either in supplement or for replacement of antibiotics [4].

Phytochemical compounds in plant extracts have attracted increasing attention for the treatment of bacterial infections. In Asia, *Morus alba* L. (Moraceae or mulberry tree) is one of the traditional medicinal plants that has been used in phytomedicine and pharmacy for the prevention of diabetes, hypertension and headaches, and has been used as a diuretic agent [5]. The fruits of the mulberry tree, i.e., the mulberries, possess supplementary health benefits due to their composition in antioxidants, namely phenolic compounds, flavonoids and anthocyanins, which are responsible for the red, purple, and/or blue colour of the fruits [6,7,8]. The major anthocyanin, cyanidin-3-O-β-d-glucopyranoside (C3G) in mulberry fruits possesses neuroprotective effects in vivo and in vitro, ischemic oxidative effect, strong α-glucoside inhibitory activity and radical scavenging activities against 2,2-diphenyl-1-picrylhdrazyl (DPPH) and superoxide anion radicals [9]. Mulberry fruits have strong antioxidant properties, which are due primarily to the presence of polyphenols, anthocyanins and flavonoids including riboflavin (vitamin B2), niacin (vitamin B3) and ascorbic acid (vitamin C) [10,11]. Some previous studies indicated that anthocyanins, rutin and polysaccharides present in mulberry extracts were the bioactive compounds preventing diabetes, cardiovascular disease, Alzheimer’s disease and obesity [8,12,13]. The mulberry extracts also exhibited antioxidant, antitumor, immunomodulation effect and especially anti-tyrosinase activity [13,14,15]. The use of mulberry as tyrosinase inhibitor has become increasingly important in the cosmetic industry due to their skin-whitening effects [11]. Besides being antinociceptive, some mulberries were reported to exhibit antibacterial activity against some Gram-positive and Gram-negative bacteria such as *Enterococcus faecalis, Escherichia coli, Klebsiella pneumoniae, Cutibacterium acnes, Pseudomonas aeruginosa, Staphylococcus epidermidis* and *Streptococcus pyogenes* [7,16]. Therefore, bioactive compounds of mulberry fruits can be explored in food, health care and cosmetic industries [11].

In Europe, mulberry trees are essentially cultivated for fruit production [6,17], whereas in East Asia, mulberry foliage is used for feeding silkworms (*Bombyx mori* L.). The three major types of fruits are white mulberry (from *Morus alba* L.), red mulberry (from *Morus rubra* L.) and black mulberry (from *Morus nigra* L.); of note, however, the colour of mulberries does not allow to determine the species of the mulberry tree [18]. White mulberry tree (*Morus alba* L.) is widely cultivated in Thailand because of healthy foliage production and easy cultivation in different environmental conditions [19]. The white mulberries from different cultivars are distributed throughout Thailand, with a major variety cultivated in northern Thailand, the Chiang Mai cultivar, which is the variety best adapted to local climate conditions and that provides mulberries of high quality.

To better characterize this variety of mulberries, antioxidant activity as well as total phenolic, flavonoid and anthocyanin content from the Chiang Mai cultivar and that of another province in Thailand, i.e., Mae Hong Son, were evaluated using different assays. The antibacterial activity of the ethanolic and aqueous extracts of white mulberry was further investigated against pathogenic enteric bacteria, as well as the effects of the extracts on bacterial growth inhibition and bacterial adhesion on intestinal epithelial cells.

## 2. Results

### 2.1. Antioxidant Activity of Mulberry Extracts

The antioxidant activity of the ethanolic and aqueous extracts of white mulberries from Chiang Mai and Mae Hong Son provinces were first evaluated by combining different complementary assays. The DPPH radical scavenging activity and ABTS radical cation decolorization activity were calculated for all extracts as IC_50_, i.e., the concentration of the extracts required to inhibit 50% of the initial free radical (Table 1). For the DPPH radical scavenging activity, the IC_50_ values varied between 0.69 mg/mL (aqueous extract from Chiang Mai) and 2.20 mg/mL (ethanolic extract from Chiang Mai), whereas the IC_50_ values ranged from 6.84 mg/mL (aqueous extract from Chiang Mai) to 15.06 mg/mL (ethanolic extract from Mae Hong Son) regarding the ABTS radical cation decolorization activity. 

Taken together, these results indicated that the white mulberry extracts exhibited various degrees of free radical cation activities as lower IC_50_ values are indicative of a higher antioxidant activity. The IC_50_ of both DPPH and ABTS were significantly higher in ethanolic extracts compared to the aqueous extracts of mulberries from Chiang Mai and Mae Hong Son provinces, respectively, which indicated a higher antioxidant activity in aqueous extracts. Similarly, it appeared that water was more efficient in extracting antioxidants from mulberries compared to ethanol. Moreover, antioxidant activities were also compared to the standard gallic acid, Trolox and ferrous sulphate (FeSO_4_) in DPPH, ABTS and FRAP assays, respectively. Aqueous extract of mulberry from Chiang Mai expressed the highest antioxidant activities of 6.74 mg gallic acid equivalent per gram extract (mg GAE/g extract), 3.34 mg Trolox equivalent antioxidant capacity (TEAC) per gram extract (mg TEAC/g extract), and 77.89 mg FeSO_4_ per gram extract (mg FeSO_4_/g extract) (Table 1). 

### 2.2. Total Phenolic, Flavonoid and Anthocyanin Content of Mulberry Extracts

Considering the content in phenolics, flavonoids and anthocyanins are the main contributors influencing antioxidant activity, they were further assayed from the different extracts of white mulberry fruits. It appeared the total phenolic content in the aqueous mulberry extracts was significantly higher than in the ethanolic extracts. Aqueous and ethanolic extracts of white mulberries from Chiang Mai or Mae Hong Son province showed a total phenolic content around 23.5 mg and 13.9 mg GAE/g, respectively (Table 2). Regarding the total flavonoid content, similar values (1.3 mg GAE/g) were obtained in aqueous extracts, whereas the content was significantly different in the ethanolic extracts with the lowest content of 0.28 mg QE/g extract from Chiang Mai and the highest of 2.54 mg QE/g from Mae Hong Son (Table 2). For anthocyanin, the content varied between 26.90 and 177.84 mg Cy-3-glc/g where it was significantly higher in the aqueous extracts (Table 2). It thus appears that the highest antioxidant activity measured with white mulberry extracts was associated with higher phenolic and anthocyanin contents following water extraction (Table 1 and Table 2).

### 2.3. Antibacterial Activity of Mulberry Extracts

The white mulberry extracts were further screened for their antibacterial activity against some key foodborne and/or enteric pathogens, namely *Escherichia coli*, *Salmonella* Typhi DMST 22842, *Shigella dysenteriae* DMST 1511, *Staphylococcus aureus* ATCC 25923 and *Vibrio cholerae*. When extracted by ethanol or water, antibacterial activities were observed against all tested bacterial species, with the growth inhibition zone ranging between 13.67 and 25.67 mm (Table 3), 24.83–29.33 mm with the positive controls (gentamicin) and no clear zone for the negative controls. Aqueous mulberry extracts appeared significantly more efficient than ethanolic extracts against *E. coli*, *S.* Typhi and *V. cholerae* (Table 3). For *S. dysenteriae*, however, similar efficiency was obtained with aqueous or ethanolic extracts, with inhibition zones comparable to those of 1 mg/mL gentamicin that was used as a positive control. In the case of *S. aureus*, a similar observation could be made for ethanolic and aqueous extracts from Mae Hong Son.

From white mulberry extracts displaying positive antibacterial activity in the agar well diffusion method, the minimum inhibitory concentration (MIC) and minimum bactericidal concentration (MBC) were determined. The results confirmed that white mulberry extracts were active against all bacterial species tested with MIC and MBC ranging from 3.91 to 125 mg/mL (Table 4). Ethanolic extract from Mae Hong Son showed the highest activity against *S. aureus* with a MIC and MBC of 3.91 mg/mL, while the aqueous extract from Chiang Mai showed the lowest activity against *E. coli* and *S.* Typhi with a MIC and MBC of 125 mg/mL. For the positive control, gentamicin could inhibit the growth of all tested bacteria with a MIC and MBC ranging from 15.63 to 31.25 µg/mL.

**Figure 1:**
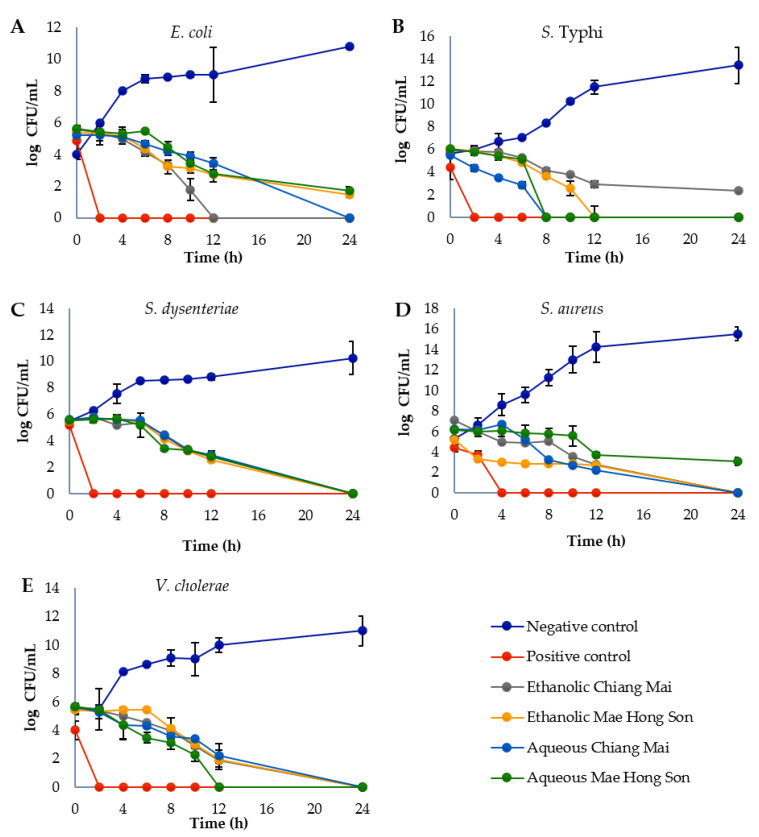
Effect of mulberry fruit extracts (1 MIC) against (**A**) *E. coli*, (**B**) *S.* Typhi, (**C**) *S. dysenteriae*, (**D**) *S. aureus,* and (**E**) *V. cholerae.* Time-kill data represented as mean ± SD (*n* = 3). DMSO was added in the negative control. Gentamicin at 1 mg/mL was used as the positive control.

The bactericidal activity of white mulberry extracts was estimated by evaluating the time course to kill the tested bacteria. The time to reach no viable cell count differed with the bacterial species tested and the different mulberry extracts (Figure 1). The ethanolic extract from Chiang Mai took 12 h to completely eradicate *E. coli* (Figure 1A). The aqueous white mulberries were the most successful at eliminating *S*. Typhi over a period of 8 h, whereas ethanolic extract from Mae Hong Son was able to completely kill the bacteria within 12 h (Figure 1B). The mulberry extracts had the slowest killing rate against *S. dysenteriae* and *S. aureus*, taking 24 h to kill the bacteria (Figure 1C,D). The aqueous extract from Mae Hong Son was able to completely kill *V. cholerae* within 12 h (Figure 1E). While both ethanolic and aqueous extracts showed some activity, the aqueous extraction of white mulberries containing more antioxidants tended to be more efficient against *S.* Typhi and *V. cholerae.*

### 2.4. Antibacterial Adhesion Activity to Intestinal Epithelial Cells of Mulberry Extracts

**Figure 2:**
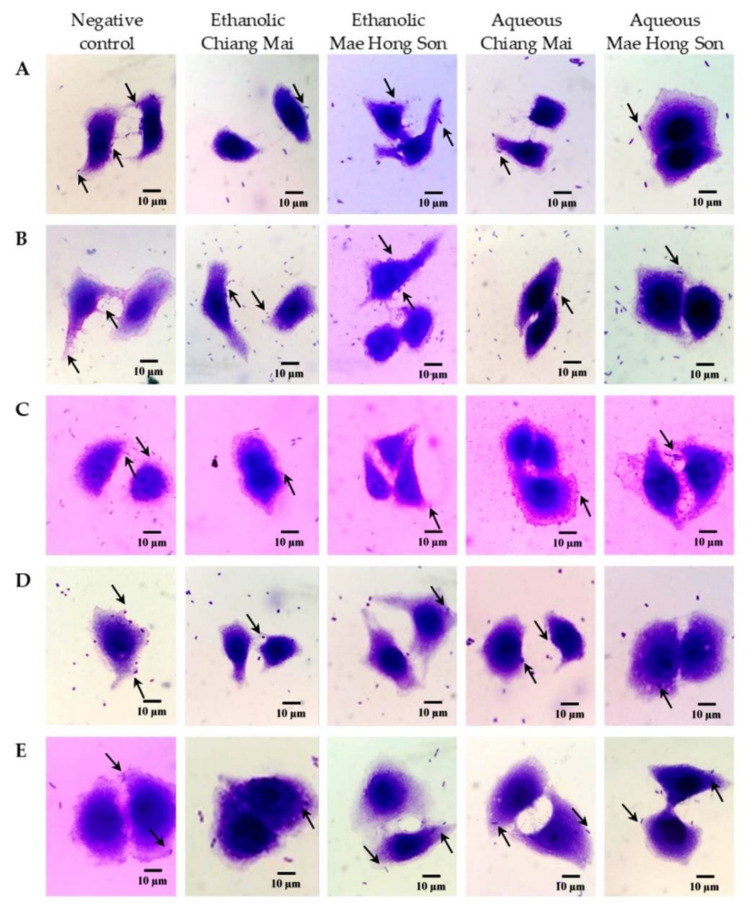
Effect of mulberry extracts on bacterial adhesion to intestinal epithelial cells. Adhesion of (**A**) *E. coli,* (**B**) *S*. Typhi, (**C**) *S. dysenteriae,* (**D**) *S. aureus* and (**E**) *V. cholerae* to Caco-2 cells after being treated with mulberry extracts as observed by microscopic examination. Arrows indicate representative adhered bacterial cell. DMSO was added in the negative control.

The antibacterial adhesion activity of white mulberry extracts was evaluated using Caco-2 cells and the results were expressed as a percentage relative to the negative control (DMSO). While the percentage of antibacterial adhesion activity ranged between 8.70 and 78.57% (Table 5 and Figure 2), the ethanolic extracts of white mulberry were the most active against adhesion of *E. coli* to Caco-2 cells, but in contrast, it showed the lowest activity in reducing the adhesion of *S. dysenteriae* (Figure 2C). Nonetheless, the aqueous mulberry extracts from both Chiang Mai and Mae Hong Son had inhibitory activity against the adhesion of *S. aureus* and *V. cholerae* and were even more efficient against *E. coli* and *S*. Typhi than ethanolic extracts. For *S. dysenteriae*, the inhibitory activity against its adhesion of the aqueous mulberry extracts from both Chiang Mai and Mae Hong Son was the lowest. Besides, for aqueous mulberry extract from Chiang Mai, the inhibitory activity against the adhesion of *V. cholerae* was slightly lower than that of ethanolic mulberry extract from Chiang Mai (Table 5 and Figure 2). 

## 3. Discussion

Mulberries are potentially a rich source of biologically active substances that could contribute to human health. White mulberries, the Chiang Mai cultivar from the Chiang Mai Province and from that of another province in Thailand, i.e., Mae Hong Son, were evaluated for their antioxidant and antibacterial activities. Polyphenolic compounds are components of antioxidants in plant foods including fruits, vegetables and grains. Therefore, it is important to measure the quantity of polyphenolic contents and to evaluate their antioxidant activity [20]. Mulberries contain a high amount of phenolic and flavonoid compounds. Fresh mulberries have a significantly higher phenolic and flavonoid content than other deep-coloured fruits [21]. The total phenolic content of mulberries was significantly higher in aqueous extracts, which differs from other results showing a higher phenolics and flavonoids content obtained from ethanolic mulberry extracts. Comparing this study to previous reports on mulberries, the total phenolic content was higher than those reported for mulberries cultivated in China [22]. The total phenolic content varied between 10.96 and 11.97 mg gallic acid equivalent (GAE)/g extract.

As revealed by a battery of complementary assays, namely DPPH, ABTS and FRAP assays, the antioxidant activity was higher in aqueous mulberry extracts. The DPPH assay is stable and generates radicals that can be dissolved in organic solvent such as methanol. Therefore, it is applicable to detect hydrophobic compounds. However, the ABTS assay is more sensitive and based on the generation of a blue/green ABTS·+, which is suitable to detect both hydrophilic and hydrophobic antioxidant compounds. Therefore, two different methods were used in this study to determine the antioxidant effects of hydrophobic and hydrophilic compound in mulberry fruit extracts, since mulberry fruits were extracted by both ethanol and water. The DPPH antioxidant activity of white mulberry extracts also appeared higher than the ones reported for mulberries cultivated in China [22]. The reducing power correlates well with antioxidant activity, and strong antioxidant activity (ABTS, DPPH) generally correlates with a high reducing power (FRAP) [23,24]. Similar to total radical absorption potentials (TRAP) [25] and the oxygen radical absorption capacity (ORAC) [26], the DPPH and ABTS [27] assays evaluated the antioxidant capacity in scavenging specific radicals that inhibit lipid peroxidation or chelating metal ions [28]. The FRAP assay, however, was developed to determine the total reducing capability of antioxidants [28].

As oxygen radical scavengers, phenolic compounds generally correlate with the antioxidant activity [29,30]. Mulberries were reported to be rich in polyphenols and possessed the ability to inhibit lipid-soluble antioxidants [31]. The content of phenolic compounds in fruits can vary and be influenced by multiple factors, such as genetic differences, environmental conditions and/or temperature [17]. Depending on the cultivars, the phenolic content of mulberries can also vary [15]. For instance, the Nakhonratchasima 60 cultivar was reported to have the highest flavonoid and phenolic acid composition, whereas the Chiang Mai cultivar had the lowest among eight major mulberries cultivated (Buriram 60, Chaingmai, Chumphon, Nakhonratchasima 60, Pikultong, Kamnanchul, Kamphaengsaen and Wavee) [19]. Besides cultivars, maturity stages can also significantly affect the phenolic content of mulberries. Phenolic contents increase from unripened to completely ripened stages, and purple mulberries generally have higher phenolic compounds than purple-red and red mulberries due to the difference in fruit maturity [32].

The content in phenolic compounds is generally well correlated with the contents in anthocyanins, including proanthocyanidins [31]. Moreover, the high content of phenolic compounds was also related to the high content of chlorogenic acids and rutin. Variation in the anthocyanin content was previously reported for mulberry cultivars in Thailand [19], which could be influenced by the production area and climate, as well as the amount of sunshine [33]; as shown with mulberries of the same cultivar, the anthocyanin content is generally higher on a sunny than on a rainy day. Even from the same species of mulberries, the different colours can indicate a different quantity of anthocyanins [6]; the purple colour in fully mature mulberries contains more anthocyanins than purple-red and red colour mulberries. Moreover, some other parameters such as the average fruit weight, total acids or soluble solid substances (e.g., sugar) can correlate with the anthocyanin content [6]. Nevertheless, the content in phenolic and anthocyanin compounds is not the only factor that can influence the antioxidant potential, since the presence of other phytochemicals, such as flavonoids and synergistic effects, can also be at play [15,20,24,34]. To date, it remains difficult to determine the contribution of each antioxidant compound considering the diversity of molecules and their different levels of interaction in fruits.

Aqueous extracts revealed a higher proportion of phenolics and anthocyanins than ethanolic extracts. The solvent use can actually reveal different kinds of phytochemical compounds in plant material. For instance, anthocyanins are water-soluble pigments in plants and will dissolve differently depending on the solvent polarity, whose abilities to dissolve or diffuse in different solvents can further affect their intrinsic bioactivity [35].

Besides antioxidant activity, the antibacterial activity of mulberry extracts was also considered. The development of antibiotic resistance in pathogenic bacteria is a major threat to public health and the use of medicinal plants can be an alternative strategy for the treatment of some bacterial infections. Mulberry extracts have antibacterial effects on *E. faecalis*, *K. pneumoniae*, *C. acnes* and *S. pyogenes* [7]. The antibacterial activity of mulberries varies depending on the bacterial strain and the extracts, which showed positive antibacterial activity in both agar well diffusion and broth dilution methods. Among the tested bacteria, *S. dysenteriae* showed maximum bacterial inhibition in the agar well method and *S*. *aureus* showed maximum bacterial inhibition of MBC by mulberry extracts, more than other tested bacteria. The effective antibacterial activity correlated with the high polyphenol and flavonoid content [36]. Similarly, the anthocyanins and flavonols of mulberries have the potential to inhibit *S. aureus, P. aeruginosa* and *E. coli* [16]. Furthermore, the antibacterial activity of polyphenolic compounds probably involves their ability to form chemical complexes with bacterial cell wall components [36], especially nucleophilic amino acids in bacterial proteins [37]. Polyphenolic compounds may also render some substrates unavailable to the bacteria cells and consequently inhibit bacterial growth [37].

Time killing can be ascribed to interactions between bacteria cells and antimicrobials present in plant extracts during their time of exposure with bacteria cells [38]. While there is no report of time-kill assay for mulberry extract, some information is available regarding other types of berries. For instance, a raspberry extract was reported to reduce the growth of *E. coli* within 10 h of treatment, whereas a cloudberry extract completely inhibited the growth of *S.* Typhimurium [39]. Bacterial cells were unable to restart new growth after 24 h [40]. For the antibacterial adhesion assay of mulberries, no data have previously been reported, and the present investigation represents the first report on the inhibition of adhesion of pathogenic enteric bacteria to intestinal epithelial cells by mulberry extracts. The adhesion ability of pathogenic enteric bacteria, such as *E. coli*, results essentially from cell surface adhesion and certain colonisation factors involved in the attachment to the intestinal cells [41]. Some phytochemicals in mulberry extract, including anthocyanins, polyphenols and flavonols, could interfere with bacterial adhesins or host cell receptors by modulating their expression [42,43,44]. In cranberry extract, proanthocyanidins inhibited the adhesion of *H. pylori* and phenolics, as well as *E. coli* and *Candida albicans* associated with urinary tract infections [45,46,47].

## 4. Materials and Methods

### 4.1. Reagents and Chemicals 

2,2-diphenyl-1-picrylhydrazil (DPPH), 2,2′-azinobis-(3-ethylbenzothiazolin-6 sulfonic acid) (ABTS), 6-hydroxy-2,5,7,8-tetramethyl-chroman-2-carboxylic acid (Trolox), gallic acid monohydrate were purchased from Sigma-Aldrich (St. Louis, MO, USA). Folin–Ciocalteu reagent, TPTZ (2,4,6-tri(2-pyridyl)-s-triazine), quercetin dehydrate were obtained from Merck (Billerica, MA, USA). Mueller–Hinton (MH) broth for antimicrobial susceptibility testing was purchased from BD (Becton, Dickinson and company, Franklin Lakes, NJ, USA). Caco-2 cell culture media, Dulbecco’s modified Eagle’s medium (DMEM) were purchased from Gibco (Grand Island, NY, USA)**.**

### 4.2. Materials

The mulberry fruits (from *M. alba* L., Chiang Mai cultivar), were collected in Chiang Mai and Mae Hong Son provinces (northern Thailand: 19°00′ N, 99°00′ E). Mulberries were dried at 60 °C in an oven and ground into powder with a blender. The samples were stored in airtight plastic bags until extraction.

### 4.3. Extraction

Aqueous and ethanolic extracts of mulberries were respectively prepared as follows [48,49]. To 200 g of mulberry powder, 2000 mL of either distilled water or 95% ethanol was added. For the water extracts, the solution was heated up to 45 °C for 3 h in a water bath (Julabo TW12, Schönwalde-Glien, Germany), where it was left at 20 °C for 72 h under orbital shaking (150 rpm) (IKA, Staufen, Germany) for the ethanolic extracts. The extracts were filtered through Whatman no. 1 filter paper. Then, the filtrate was concentrated under a vacuum at 45 °C using a rotary evaporator (Heidolph, Schwabach, Germany) and freeze-dried (LABCONCO, MO, USA) by lyophilisation. All crude extracts after freeze-drying were stored at –20 °C until used for experimentation.

### 4.4. Antioxidant Activities 

#### 4.4.1. DPPH Radical Scavenging Assay

The radical scavenging activity was measured using 2,2-diphenyl-1-picrylhydrazil (DPPH) assay [50] with some modifications. Methanolic solutions of DPPH (0.1 mM) and mulberry extracts with various concentrations (0.1–10.0 mg/mL) were prepared. An aliquot of 0.5 mL of each sample was mixed with 1.5 mL of DPPH solution and incubated for 20 min in the dark at room temperature. The absorbance was measured at 517 nm using a spectrophotometer (Thermo Scientific GENESYS 20, Leicestershire, UK) and the results were presented as IC_50_, which is the necessary concentration to inhibit 50% of free radical activity. The radical scavenging activity was calculated according to the following equation:

$$\mathrm{DPPH}\;\mathrm{radical}\;\mathrm{scavenging}\;\mathrm{activity}\;\left(\%\right)=\frac{A\;\mathrm{control}-A\;\mathrm{treatment}}{A\;\mathrm{control}}\times 100$$

In the DPPH assay, gallic acid was used as a positive control. The radical scavenging activity of mulberry fruit extracts was compared to standard gallic acid and the results were presented as milligram of gallic acid equivalent per gram extract (mg GAE/g extract).

#### 4.4.2. ABTS Radical Cation Decolorization Assay

The radical cation decolorization activity was measured using 2,2′-azinobis-(3-ethylbenzothiazolin-6-sulfonic acid) (ABTS^.+^) assay [51,52] with minor modifications. An equal volume of ABTS^.+^ solution (7 mM) and potassium persulfate solution (2.45 mM) were mixed in the dark at room temperature for 12 to 16 h to generate ABTS^.+^ radical. The ABTS^.+^ solution was diluted with distilled water to obtain an absorbance of 0.700 ± 0.020 at 734 nm before use. The mulberry extract (5 µL) or Trolox standard was mixed with the diluted ABTS^.+^ solution (195 µL) and incubated for 10 min. The absorbance was measured at 734 nm using a microplate reader (DYNEX SPECTRA MR Microplate Spectrophotometer, VA, USA). The radical cation decolorization activity was compared with the Trolox equivalent antioxidant capacity (TEAC) and the results were presented as IC_50_, which is the concentration of the extract necessary to inhibit 50% of free radical activity. The percentage of inhibition was calculated according to the following equation:

$$\mathrm{ABTS}\;\mathrm{radical}\;\mathrm{cation}\;\mathrm{decolorization}\;\mathrm{activity}\;\left(\%\right)=\frac{A\;\mathrm{control}-A\;\mathrm{treatment}}{\;A\;\mathrm{control}}\times 100$$

In part of the ABTS assay, Trolox was used as a positive control. The radical scavenging activity of mulberry fruit extracts was compared to that of standard Trolox and the results were presented as milligram Trolox equivalent antioxidant capacity (TEAC) per gram extract (mg TEAC/g extract).

#### 4.4.3. Ferric Reducing Antioxidant Power (FRAP) Assay

Ferric reducing antioxidant power was performed using reductants in a redox-linked colorimetric method [53]. The FRAP reagent included 300 mM acetate buffer pH 3.6, 20 mM ferric chloride solution, 10 mM TPTZ (2,4,6-tri(2-pyridyl)-s-triazine) in 40 mM hydrochloric acid and distilled water. These were combined and kept in a water bath at 37 °C. The mulberry extract (0.5 mL) was mixed with 1.5 mL of FRAP reagent. After incubation for 15 min in the dark at room temperature, the absorbance was measured at 593 nm using a spectrophotometer (Thermo Scientific GENESYS 20, Leicestershire, UK).

In FRAP assay, ferrous sulphate (FeSO_4_) was used as a positive control. The ferric reducing antioxidant activity was calculated through the ferrous sulphate (FeSO_4_) calibration curve within a range of 10 to 100 µg/mL (R^2^ = 0.9998). The results were presented as milligram ferrous sulphate (FeSO_4_) per gram extract (mg FeSO_4_/g extract).

### 4.5. Determination of Total Phenolic Flavonoid and Anthocyanin Content

#### 4.5.1. Total Phenolic Content

Total phenolic content was evaluated using the Folin–Ciocalteu method [53]. Mulberry extract (250 µL) was mixed with 125 µL of 50% Folin–Ciocalteu reagent, followed by 1.25 mL of distilled water and 250 µL of 95% ethanol. After 5 min, the 250 µL of 5% *w*/*v* sodium carbonate solution was added and incubated at room temperature for 1 h. Afterward, the absorbance was measured at 725 nm using a spectrophotometer (Thermo Scientific GENESYS 20, Leicestershire, UK). The total phenolic content was calculated through the gallic acid calibration curve within a range of 10 to 100 µg/mL (R^2^ = 0.9985). The results were presented as milligram gallic acid equivalents (GAE) per gram extract (mg GAE/g extract).

#### 4.5.2. Total Flavonoid Content

Total flavonoid content was determined using the aluminium chloride colorimetric method [50]. Mulberry extract (500 µL) was mixed with 100 µL of 10% aluminium chloride, followed by 1.5 mL of methanol, 100 µL of 1 M potassium acetate and 2.8 mL of distilled water. After 30 min at room temperature, the absorbance was measured at 415 nm using a spectrophotometer (Thermo Scientific GENESYS 20, Leicestershire, UK). The total flavonoid content was calculated through the quercetin calibration curve within a range of 7.812 to 125 µg/mL (R^2^ = 0.9999). The results were presented as milligram quercetin equivalent (QE) per gram extract (mg QE/g extract).

#### 4.5.3. Determination of Total Anthocyanin Content

Total anthocyanin content was evaluated using the pH differential method [54]. Two dilutions of the sample were prepared for pH 1.0 using 0.025 M potassium chloride buffer and the other sample was prepared for pH 4.5 using 0.4 M sodium acetate buffer. The mulberry extract (20 µL) was diluted 10 times to a final volume of 200 µL. After incubation for 15 min at room temperature, the absorbance was measured at 510 and 700 nm using a microplate reader (DYNEX SPECTRA MR Microplate Spectrophotometer, VA, USA). Total anthocyanin content was calculated using the following formula and presented as milligram cyanidin 3-glucoside equivalent per gram extract (mg Cy-3-glc/g extract).

A = (A_510_ − A_700_) _pH1.0_ − (A_510_ − A_700_) _pH4.5_

$$\mathrm{TA}=\frac{A\times \mathrm{MW}\times \mathrm{DF}\times 1000}{\varepsilon \times 1}$$

where A is the absorbance, MW is the molecular weight (g/mol) = 449.2 g/mol for Cy-3-glc, DF is the dilution factor, ε is the extinction coefficient (*L* × cm^−1^ × mol^−1^) = 26,900 for Cy-3-glc, where *L* (path length in cm) = 1.

### 4.6. Microorganism

Pathogenic enteric bacteria, *Escherichia coli* and *Vibrio cholerae* were kindly obtained from the Microbiology Section, Department of Medical Technology, Faculty of Associated Medical Science, Chiang Mai University, Chiang Mai, Thailand. *Salmonella* Typhi DMST 22842, *Shigella dysenteriae* DMST 1511 and *Staphylococcus aureus* ATCC 25923 were obtained from SCB 2711 Microbiology Laboratory, Chiang Mai University, and used for the determination of antibacterial activity.

### 4.7. Antibacterial Activity of Mulberry Extracts

#### 4.7.1. Determination of Antibacterial Activity of Mulberry Extracts by Agar Well Diffusion Method

The antibacterial activity of mulberry extracts was evaluated by the agar well diffusion method [55]. A bacterial suspension was used to adjust the turbidity with the MacFarland standard solution no. 0.5 and was then swabbed on Mueller–Hinton (MH) agar plates. The mulberry extracts were dissolved in DMSO to obtain a concentration of 500 mg/mL. Wells with an 8 mm diameter were generated on the surface of the agar with a sterile cork borer and 100 µL of extract was transferred into the wells. A standard well with 1 mg/mL gentamicin and DMSO was used as a positive and a negative control, respectively. Plates were incubated at 37 °C for 24 h and the antimicrobial activity was determined by measuring the diameter of the inhibition zone against the tested bacteria species.

#### 4.7.2. Determination of Minimum Inhibitory Concentration (MIC) and Minimum Bactericidal Concentration (MBC) of Mulberry Extracts

The MIC was observed and defined as the lowest concentration of the extracts that visibly inhibited bacterial growth [56]. Mulberry extract was diluted by twofold serial dilution. The extract (200 µL) was transferred to test tubes of 200 µL with sterile MH broth. After that, a twofold serial dilution was generated at the concentrations of 250, 125, 62.5, 31.25, 15.63, 7.81, 3.91 and 1.95 mg/mL. The bacterial inoculum was prepared in comparison with McFarland standard no. 0.5 and inoculated into test tubes containing 200 µL of the various concentrations of the extracts. For gentamicin, an initial concentration of 500 µg/mL was used and diluted to concentrations of 250, 125, 62.5, 31.25, 15.63, 7.81 and 3.91 µg/mL. Then, the bacterial culture was added into the tubes. All test tubes were incubated at 37 °C for 24 h.

In order to determine the MBC, an aliquot from all tubes from which there was no visible bacterial growth was streaked on MH agar plates, and the plates were incubated at 37 °C for 24 h. The MBC endpoint was defined as the lowest concentration of the extract, at which 99.9% of the final bacteria inoculum was killed [57].

#### 4.7.3. Time-Kill Assay of Bacteria by Mulberry Extracts

Mulberry extracts at 1 MIC were tested for growth inhibition by a time-kill assay. The bacteria (1.5 × 10^5^ CFU/mL) were incubated with mulberry extracts in MH broth. Bacterial growth was quantified after 0, 2, 4, 6, 8, 10, 12 and 24 h incubation at 37 °C. At each sample time, a 100 µL aliquot was diluted in 0.85% saline and the diluted sample (100 µL) was spread onto MH agar plates. After incubation at 37 °C for 24 h, colony counts were evaluated and compared to the starting inoculum [58].

### 4.8. Cell Culture

Caco-2 human colorectal carcinoma cells were cultured in Dulbecco’s modified Eagle’s medium (DMEM) containing 10% heat-inactivated foetal bovine serum (FBS), 1% penicillin-streptomycin (100 U/mL penicillin, and 100 µg/mL streptomycin) and maintained at 37 °C in a 5% CO_2_ incubator.

### 4.9. Determination of Cell Toxicity by Mulberry Extracts

Prior to bacterial adhesion to intestinal epithelial cells, the potent toxicity of mulberry extracts on Caco-2 cells was evaluated in vitro using a 3-(4,5-dimethylthiazol-2-yl)-2,5 diphenyltetrazolium bromide (MTT) assay with some modifications [59]. The Caco-2 cells were grown in 96-well plates at a density of 1 × 10^5^ cells/mL (100 μL). After incubation at 37 °C in a 5% CO_2_ incubator for 24 h, the ethanolic (0.625 mg/mL) and aqueous (1.25 mg/mL) mulberry extracts at 100 µL were added to the wells and incubated for 24 h. Then, an MTT solution (2 mg/mL in phosphate buffer saline) was added to each well and the plates were incubated at 37 °C for 4 h. After incubation, the medium from the wells were removed and DMSO (200 μL) was added to solubilize the formed formazan crystals. The absorbance was measured on a microplate reader using a test wavelength of 540 nm and a reference wavelength of 630 nm.

### 4.10. Determination of Antibacterial Adhesion by Mulberry Extracts

The efficacy of the mulberry extracts on Caco-2 cells for the adhesion to the tested bacteria was determined using the antibacterial adhesion assay with slight modifications [44,60]. The cell suspension with 2 × 10^3^ cells was transferred to microscope cover glasses, placed in 6-well plates then incubated in 5% CO_2_ at 37 °C for 24 h. Bacterial suspension was adjusted to 1 × 10^8^ CFU/mL. Afterward, Caco-2 cells were infected with 1 mL of bacterial suspension and treated with 1 mL of mulberry extract. After incubation for 1 h, the supernatant was removed from the wells. The cells were washed with PBS and fixed with methanol for 5 min. Methanol was discarded and fixed cells were stained with 0.38% *w*/*v* Giemsa stain for 15 min. The microscope cover glasses were removed, and the excess stain was investigated under a light microscope (OLYMPUS CX31, PA, USA). The number of cells to which bacteria had adhered was counted. The percentage of antibacterial adhesion was calculated and compared to the negative control using the formula:

$$\mathrm{Percentage}\;\mathrm{of}\;\mathrm{anti}-\mathrm{bacterial}\;\mathrm{adhesion}\;\left(\%\right)=\frac{N\;\mathrm{control}-N\;\mathrm{treatment}}{\;N\;\mathrm{control}}\times 100$$

### 4.11. Statistical Analysis

The statistical analysis of the data was executed using SPSS 23.0 for Windows. All experiments were performed in triplicate and presented as mean ± standard deviations using one-way analysis of variance (ANOVA).

## 5. Conclusions

As a whole, the aqueous extracts of white mulberries appear to be a good source of antioxidants as determined by the DPPH, ABTS, and FRAP assays, with a high phenolic and anthocyanin content. Furthermore, white mulberry extracts appear to be a good antimicrobial agent against pathogenic enteric bacteria causing gastrointestinal tract infections. While these results support the use of mulberries as a potential supplementary food for the prevention of diseases generated by free radicals or to treat bacterial infections, further investigations are still needed. In fact, the efficiency of fruit extracts can be mitigated in vivo in the gastrointestinal tract as phytochemicals can be partly metabolised by the gut microbiota. For instance, the proportion of new metabolites generated and the activity of anthocyanins, polyphenols and/or flavonoids can be further affected and vary within the digestive environment. Besides animal models, the use of simulated digestive models can help to investigate these aspects and to ultimately evaluate their benefit for human health [61,62,63].

## Figures and Tables

**Table 1:** Evaluation of antioxidant activities of mulberry extracts from DPPH, ABTS and FRAP assays.

Extraction	Province of Origin	DPPH		ABTS		FRAP (mg FeSO_4_/g Extract)
		IC_50_ (mg/mL)	Antioxidant Activity (mg GAE/g Extract)	IC_50_ (mg/mL)	Antioxidant Activity (mg TEAC/g Extract)	
Ethanolic	Chiang Mai Mae Hong Son	2.20 ± 0.16 ^a^	1.77 ± 0.46 ^a^	10.09 ± 0.05 ^b^	2.13 ± 0.02 ^ab^	52.67 ± 1.34 ^a^
		1.53 ± 0.18 ^b^	2.61 ± 0.39 ^a^	15.06 ± 0.88 ^c^	1.78 ± 0.03 ^a^	62.18 ± 2.05 ^ab^
Aqueous	Chiang Mai	0.69 ± 0.04 ^c^	6.74 ± 0.66 ^b^	6.84 ± 0.59 ^a^	3.34 ± 0.36 ^c^	77.89 ± 1.11 ^c^
	Mae Hong Son	1.00 ± 0.29 ^c^	4.22 ± 1.53 ^ab^	10.53 ± 0.37 ^b^	2.54 ± 0.57 ^b^	67.51 ± 2.51 ^bc^

Each value in the table is represented as mean ± SD (n = 3); ^a,b,c^ indicate significant difference (*p* < 0.05).

**Table 2:** Total phenolic, flavonoid and anthocyanin content of mulberry extracts.

Extraction	Province of Origin	Total Phenolic Content (mg GAE/g Extract)	Total Flavonoid Content (mg QE/g Extract)	Total Anthocyanin Content (mg Cy-3-glc/g Extract)
Ethanolic	Chiang Mai	13.97 ± 0.71 ^a^	0.28 ± 0.05 ^a^	26.90 ± 1.95 ^a^
	Mae Hong Son	13.95 ± 2.85 ^a^	2.54 ± 0.11 ^b^	35.90 ± 1.18 ^b^
Aqueous	Chiang Mai	23.77 ± 1.96 ^b^	1.24 ± 0.17 ^c^	143.61 ± 2.95 ^c^
	Mae Hong Son	23.36 ± 2.87 ^b^	1.43 ± 0.03 ^c^	177.84 ± 3.54 ^d^

Each value in the table is represented as mean ± SD (n = 3); ^a,b,c,d^ indicate significant difference (*p* < 0.05).

**Table 3:** Antibacterial activity of mulberry extracts against some pathogenic enteric bacteria.

Extraction	Province of Origin	Inhibition Zone Diameter (mm)				
		*E. coli*	*S.* Typhi	*S. dysenteriae*	*S. aureus*	*V. cholerae*
Ethanolic	Chiang Mai	13.83 ± 0.76 ^a^	13.67 ± 0.58 ^a^	20.83 ± 0.76 ^a^	17.83 ± 0.76 ^a^	15.00 ± 0.50 ^a^
	Mae Hong Son	15.50 ± 0.50 ^ab^	16.50 ± 0.50 ^b^	25.67 ± 0.58 ^c^	23.50 ± 0.50 ^c^	16.00 ± 00 ^ab^
Aqueous	Chiang Mai	16.17 ± 0.76 ^b^	16.67 ± 0.76 ^b^	20.00 ± 0.00 ^a^	20.83 ± 0.76 ^b^	16.33 ± 0.76 ^ab^
	Mae Hong Son	20.00 ± 0.00 ^c^	17.50 ± 0.50 ^b^	24.00 ± 0.00 ^c^	22.33 ± 0.58 ^bc^	17.17 ± 0.29 ^b^
Positive control		28.83 ± 0.76 ^d^	27.50 ± 0.87 ^c^	24.83 ± 0.76 ^bc^	29.33 ± 0.58 ^d^	29.33 ± 0.58 ^c^

Each value in the table is represented as mean ± SD (n = 3) using 500 mg/mL mulberry extracts. Results are expressed as diameter (in mm) of the inhibition zone as determined by the well diffusion method. ^a,b,c,d^ indicate significant difference (*p* < 0.05). Gentamicin at 1 mg/mL was used as the positive control.

**Table 4:** Antibacterial activity of mulberry fruit extracts as determined by minimum inhibitory concentration (MIC) and minimum bactericidal concentration (MBC).

Extraction	Province of Origin	Concentration of Mulberry Extract (mg/mL)									
		*E. coli*		*S.* Typhi		*S. dysenteriae*		*S. aureus*		*V. cholerae*	
		MIC	MBC	MIC	MBC	MIC	MBC	MIC	MBC	MIC	MBC
Ethanolic	Chiang Mai	62.5	62.5	15.63	31.25	31.25	31.25	31.25	31.25	15.63	62.5
	Mae Hong Son	31.25	31.25	31.25	31.25	15.63	15.63	3.91	3.91	15.63	15.63
Aqueous	Chiang Mai	125	125	125	125	31.25	62.5	62.5	62.5	31.25	62.5
	Mae Hong Son	62.5	62.5	31.25	31.25	62.5	62.5	7.81	15.63	15.63	15.63

**Table 5:** Effect of mulberry fruit extracts on antibacterial adhesion of pathogenic enteric bacteria to intestinal epithelial cells.

Mulberry Fruit Extracts	Province of Origin	% Inhibition of Bacterial Adhesion				
		*E. coli*	*S.* Typhi	*S. dysenteriae*	*S. aureus*	*V. cholerae*
Ethanolic	Chiang Mai	78.57 ± 2.66 ^d^	55.24 ± 3.19 ^b^	43.48 ± 3.00 ^d^	32.01 ± 2.65 ^a^	44.34 ± 1.69 ^b^
	Mae Hong Son	9.52 ± 1.32 ^a^	28.63 ± 4.94 ^a^	8.70 ± 1.02 ^a^	43.88 ± 1.66 ^b^	16.04 ± 1.07 ^a^
Aqueous	Chiang Mai	73.81 ± 1.07 ^c^	75.00 ± 4.33 ^c^	21.74 ± 2.89 ^b^	58.27 ± 3.00 ^c^	42.45 ± 3.30 ^b^
	Mae Hong Son	58.73 ± 0.11 ^b^	66.53 ± 1.43 ^c^	34.78 ± 3.80 ^c^	58.27 ± 4.29 ^c^	58.49 ± 1.79 ^c^

Each value in the table is represented as mean ± SD (n = 3); ^a,b,c,d^ indicate significant difference (*p* < 0.05).

## Data Availability

The data presented in this study are available on request from the corresponding author.
